# Root flavonoids are related to enhanced AMF colonization of an invasive tree

**DOI:** 10.1093/aobpla/plaa002

**Published:** 2020-01-11

**Authors:** Yingchun Pei, Evan Siemann, Baoliang Tian, Jianqing Ding

**Affiliations:** 1 School of Life Sciences, Henan University, Kaifeng, Henan, China; 2 State Key Laboratory of Crop Stress Adaptation and Improvement, Henan University, Kaifeng, Henan, China; 3 Biosciences Department, Rice University, Houston, TX, USA

**Keywords:** Biomass, Chinese tallow tree, flavonoids, invasive population, secondary metabolism

## Abstract

Arbuscular mycorrhizal fungi (AMF) are important mutualistic microbes in soil, which have capacity to form mutualistic associations with most land plants. Arbuscular mycorrhizal fungi play an important role in plant invasions and their interactions with invasive plants have received increasing attention. However, the chemical mechanisms underlying the interactions of AMF and invasive plants are still poorly understood. In this study we aim to test whether root secondary chemicals are related to enhanced AMF colonization and rapid growth in an invasive tree. We conducted a common garden experiment in China with Chinese tallow tree (*Triadica sebifera*) to examine the relationships among AMF colonization and secondary metabolites in roots of plants from introduced (USA) and native (China) populations. We found that AMF colonization rate was higher in introduced populations compared to native populations. Roots of plants from introduced populations had lower levels of phenolics and tannins, but higher levels of flavonoids than those of plants from native populations. Flavonoids were positively correlated with AMF colonization, and this relationship was especially strong for introduced populations. Besides, AMF colonization was positively correlated with plant biomass suggesting that higher root flavonoids and AMF colonization may impact plant performance. This suggests that higher root flavonoids in plants from introduced populations may promote AMF spore germination and/or attract hyphae to their roots, which may subsequently increase plant growth. Overall, our results support a scenario in which invasive plants enhance their AMF association and invasion success via genetic changes in their root flavonoid metabolism. These findings advance our understanding of the mechanisms underlying plant invasion success and the evolutionary interactions between plants and AMF. Understanding such mechanisms of invasive plant success is critical for predicting and managing plant invasions in addition to providing important insights into the chemical mechanism of AMF–plant interactions.

## Teaser

In this study we found that the arbuscular mycorrhizal fungi (AMF) colonization rate was higher in introduced populations compared to native populations. Roots of plants from introduced populations had higher levels of flavonoids and the concentration of flavonoids in roots was positively correlated with AMF colonization, especially strong for introduced populations. Furthermore, AMF colonization was positively correlated with plant biomass. These findings suggest that higher root flavonoids in plants from introduced populations may promote AMF spore germination and/or attract hyphae to their roots, which may subsequently increase plant growth.

## Introduction

Plant invasions can damage the ecological environment (Tanveer *et al.* 2018) by reducing the diversity or abundance of native plant and animal communities (Moroń *et al.* 2009; Stefanowicz *et al.* 2017). In order to clarify the mechanisms underlying plant invasions, there have been an increasing number of studies on the role of below-ground biota, such as the effects of soil microbes on invasive plants (Rout and Callaway 2012; Li *et al.* 2017; Verbeek and Kotanen 2019). Arbuscular mycorrhizal fungi (AMF), an important group of symbiotic microbes (van Kleunen *et al.* 2018), have been found to play a role in plant invasions (Richardson *et al.* 2010; van Kleunen *et al.* 2018; Yong *et al.* 2018). However, it is not known why invasive plants or introduced populations of invasive plants often have higher rates of mycorrhizal colonization than native plants or native populations of invasive plants, respectively.

Arbuscular mycorrhizal fungi build symbiotic relationships with >80 % of terrestrial plants, including many invasive plants (Reinhart and Callaway 2006; Zdenka *et al.* 2013; Horn *et al.* 2017; Mello and Balestrini 2018). They typically benefit their host plants by promoting soil nutrient mobilization and absorption (Rillig 2010; Datta and Kulkarni 2014; Bunn *et al.* 2015; Kim *et al.* 2015; Jiang *et al.* 2018; Zhang *et al.* 2019). At present, an increasing number of studies have demonstrated that AMF can have an important role in plant invasion success (Dawkins and Esiobu 2017; Zhang *et al.* 2018). The enhanced mutualisms hypothesis indicates that invasive species can alter the AMF community and receive greater benefits from them compared to co-occurring native plants, which may facilitate their invasion (Reinhart and Callaway 2006) by increasing survival, growth rate and/or competitiveness (Sun and He 2010; Lekberg *et al.* 2013; Zhang *et al.* 2017). One study found that plant invasions can increase the diversity of AMF by comparing uninvaded and invaded sites in Hawaii (Gomes *et al.* 2018). Another study found that the invasive plants, *Ambrosia artemisiifolia* and *Bidens pilosa*, have higher AMF colonization rates than the native plant *Setaria viridis* when they are planted together (Zhang *et al.* 2018). Furthermore, the invasive Eurasian forbs knapweed (*Centaurea stoebe*), leafy spurge (*Euphorbia esula*) and Canada goldenrod (*Solidago canadensis*) each benefit in competition with native plants when mycorrhizae are present (Sun and He 2010; Lekberg *et al.* 2013). The invasive Chinese tallow tree (*Triadica sebifera*) has also been found to gain more benefits from mycorrhizal associations than co-occurring native trees in a range of soil fertilities (Nijjer *et al.* 2004, 2008; Paudel *et al.* 2014). Hence, it can be inferred that the AMF may play an important role in the establishment and spread of invasive plants (Sielaff *et al.* 2019). Although we have a wealth of evidence showing that invasive plants and introduced populations of invasive plants often benefit more from AMF associations than native species or native populations, respectively, we know little about what drives these higher rates of mycorrhizal colonization that underlie these high colonization rates and benefits.

Indeed, some studies have explored the chemical mechanisms that drive differences in mycorrhizal colonization rates for plants in general. For instance, a study on the root exudates from tomato found an unknown active factor, which is a methanol-soluble compound but not the strigolactone analog GR24, stimulates AMF growth and branching (Sun *et al.* 2012). A study on legumes showed that flavonoids play an important role in signalling, establishment and regulation of mycorrhizal endosymbiosis (Singla and Garg 2017), as well as playing a role in anti-herbivore defence (Xiao *et al.* 2019) and antioxidant activity (Ma *et al.* 2017). This finding is supported by an RNAi silencing study, which found that mycorrhizal colonization is affected by flavonoids and polyamines in soybeans (Salloum *et al.* 2018). In addition, research on the effect of the essential oil from aromatic lavender (*Lavandula stoechas*) on two mycorrhizal species indicated that it was beneficial for the colonization of *Septoglomus deserticola* and *Rhizophagus intraradices* (Hassiotis and Orfanoudakis 2018). Other studies have demonstrated that some flavonoids can induce AMF spore germination and hyphal branching, potentially increasing the colonization rate on plant roots (Akiyama *et al.* 2005; Liu *et al.* 2014). For example, the flavonoid apigenin is able to enhance hyphal branching and root colonization at 0.5 μM concentration (Scervino *et al.* 2006). A study on melon found that flavonoids are involved in the regulation of AMF infection in its roots (Akiyama *et al.* 2002). However, there are no investigations of the regulation of AMF by secondary chemicals in invasive plants or the potential role of differences in secondary chemicals, especially root flavonoids, of introduced vs. native populations of invasive plants on their AMF associations.

Chinese tallow tree (*T. sebifera*) is a deciduous tree that is originally from China (Pattison and Mack 2008). It is introduced to USA in the late 18th century where it has become invasive (Pile *et al.* 2017). Previous studies showed that plants from introduced populations have higher AMF colonization (Yang *et al.* 2013, 2015c), more rapid growth and greater competitiveness (Huang *et al.* 2012b; Siemann *et al.* 2017) and higher foliar flavonoids than native populations (Wang *et al.* 2012). In this study, we investigated whether higher flavonoids in roots of *T. sebifera* plants from introduced populations are correlated with higher AMF colonization rates, which contribute to their rapid growth.

## Materials and Methods

### Seeds collection

We collected seeds of *T. sebifera* from 12 populations in southern China (native populations) and 10 populations across the south-eastern USA (invasive populations) in November 2015 (Table 1). These populations included the likely source and recipient populations from the two major North American introduction events (Dewalt *et al.* 2011). We hand-collected seeds from 5 to 10 trees in every population. We removed the waxy layer around these seeds by soaking them in water with laundry detergent (Huang *et al.* 2012a), then we rinsed them and put them in the refrigerator (4 °C) in wet sand. After 30 days, we planted them in sterile garden soil.

**Table 1.** The geographical coordinates of *Triadica sebifera* populations from the native (China, 12 populations) and introduced (USA, 10 populations) ranges in this study.

ID	Site of seed collection	Latitude	Longitude
Native populations (in China)			
CH-DW	Dawu, Hubei	31°28′N	114°16′E
CH-GL	Guilin, Guangxi	25°04′N	110°18′E
CH-HeS	Hengyang, Hunan	27°14′N	112°46′E
CH-HF	Hefei, Anhui	31°50′N	117°09′E
CH-HuS	Huangshan, Anhui	30°00′N	117°59′E
CH-LiA	Lin’an, Zhejiang	30°47′N	120°03′E
CH-ML	Miluo, Hunan	28°53′N	113°12′E
CH-NJ	Nanjing, Fujian	24°42′N	117°3′3E
CH-WX	Wuxi, Jiangsu	31°36′N	120°14′E
CH-YS	Yangshan, Guangdong	24°35′N	112°41′E
CH-ZS	Zhangshu, Jiangxi	28°02′N	115°25′E
CH-YT	Yingtan, Jiangxi	28°19′N	117°03′E
Introduced populations (in USA)			
US-AL-1	Tillman’s Corner, AL	30°35′N	88°09′W
US-FL-4	Callahan, FL	30°35′N	81°47′W
US-GA-1	Hutchinson Island, GA	32°06′N	81°06′W
US-GA-2	Sapelo Island, GA	31°23′N	81°15′W
US-LA-1	Lake Charles, LA	30°14′N	93°09′W
US-LA-5	Pumpkin Center, LA	30°28′N	90°32′W
US-SC-1	Limehouse, SC	32°09′N	81°06′W
US-TX-2	La Marque, TX	29°22′N	95°02′W
US-TX-4	Lynchburg, TX	29°47′N	95°02′W
US-TX-5	Port Arthur, TX	29°53′N	94°02′W

### Soil

We mixed together (1:1 volume) commercial river sand and soil which were collected from a field at Henan University (Kaifeng, Henan province, China) in which maize had been grown in the previous season. We filled 198 plastic pots (height: 15 cm, upper diameter: 19 cm, bottom diameter: 12 cm) each with 1.5 kg of this soil mixture.

### Experimental design

To test the relationship between plant growth and AMF colonization, we carried out a common garden experiment from June to October in 2016 at Henan University. In July, when seedlings had reached the four-leaf stage, nine seedlings of each population individually were transplanted into the pots with the sand/soil mix. These seedlings were protected with nylon mesh (40 openings per inch) from herbivory and placed in an open-sided greenhouse, and then they were watered daily.

After 105 days, these seedlings were clipped at ground level before we collected soil samples to measure hyphal density. Then, we carefully washed the roots from the soil and took a subsample of fine roots to assess AMF colonization rate. Following that, we dried (40 °C for 48 h) and then weighed above-ground and below-ground biomass, separately.

### AMF colonization

We cleared fresh fine roots with 10 % KOH for 60 min at 90 °C, acidified them with 2 % HCl for 5 min and then stained them for 30 min at 90 °C with 0.05 % trypan blue following published protocols (Nijjer *et al.* 2008). We mounted 30 1-cm fine root segments from each plant onto slides. Arbuscular mycorrhizal fungi colonization rate was estimated by using the gridline intersect method with 300 intersection points per plant (Brundrett *et al.* 1995).

### Hyphal density

We estimated hyphal length in soil following a modification of published methods (Hanssen *et al.* 1974; Johansen *et al.* 1992). We blended 2 g soil and 50 mL of Deionized water for 30 s at high speed (10 000 rpm). We transferred each soil solution into a 500-mL beaker with 200 mL of Deionized water and mixed it with a magnetic stirrer (900 rpm). We let the solution rest for 30 s, then took 5 mL of this solution from a depth of 1 cm from the top and filtered it through a Millipore filters (0.45 μm) three times per soil sample (three separate filters). We covered the filters for 5 min with 0.05 % trypan blue. We dried them and recorded the presence of hyphae at 25 intersections of a 1-mm grid at ×200 magnification. We calculated hyphal density by the formula: Hyphal density (m g^−1^ dry soil) = 0.14399 × number of crossings (Jakobsen *et al.* 1992; Gao *et al.* 2016).

### Total soluble sugars

We ground dried roots with a ball mill (Hengao HMM-400A, Tianjin Hengao Technology Development Co., Ltd). We extracted total soluble sugars from 100 mg root samples using 95 % ethanol and determined the concentrations of soluble sugar with the colourimetry of sulfuric acid-anthrone method (Yemm and Willis 1954).

### Secondary metabolites

We measured the concentration of total phenolics by the Folin-Ciocaileu colourimetric method (Stankovic *et al.* 2011) and the concentration of total flavonoids by the modified method of aluminium nitrate colourimetric (Medini *et al.* 2014; Li *et al.* 2019). We measured total tannin with the vanillin-hydrochloric acid method (Price *et al.* 1978).

### Data analysis

We used analysis of variance (ANOVA) to test the dependence of AMF colonization, hyphal density, total soluble sugars and secondary compounds on population origin (fixed factor) and population nested in origin as a random factor (proc mixed, SAS 9.4). We used ANOVA to test the dependence of AMF colonization on root flavonoids, population origin and their interaction as fixed terms and population nested in origin as a random factor. We used Pearson correlations to examine the relationships among AMF colonization and plant mass (the total mass of above-ground and below-ground biomass), the root concentrations of polyphenols, tannins, soluble sugars and flavonoids (proc corr, SAS 9.4). Because native and introduced population plants differed significantly in AMF colonization and root flavonoids and visual inspection indicated different relationships between root flavonoids and AMF colonization for introduced vs. native populations, we performed separate Pearson correlations for them as well.

## Results

### AMF of native and introduced populations

**Figure 1.**
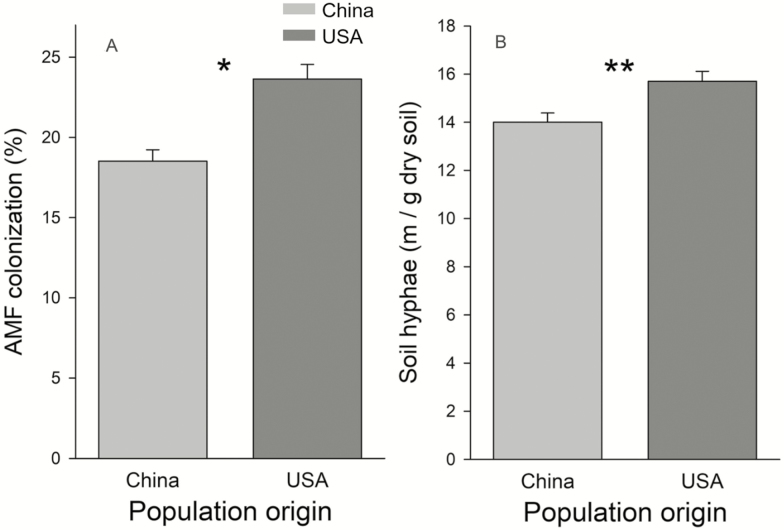
(A) AMF colonization on roots and (B) fungal hyphae in soils associated with *T. sebifera* plants from native (China) or introduced (USA) populations. Difference of native (China) and introduced (USA): *P < 0.05; **P < 0.01.

Plants from introduced populations had higher levels of AMF colonization on their roots than native populations (Fig. 1A; *F*_1, 20_ = 5.18, *P* = 0.0341) and a greater density of hyphae in soil associated with their roots (Fig. 1B; *F*_1, 20_ = 9.18, *P* = 0.0060). Populations varied in their AMF colonization (*Z* = 2.62, *P* = 0.0044) but not density of hyphae in soil (*Z* = 0.61, *P* = 0.5431).

### Concentrations of metabolite in the roots of native and introduced populations

**Figure 2.**
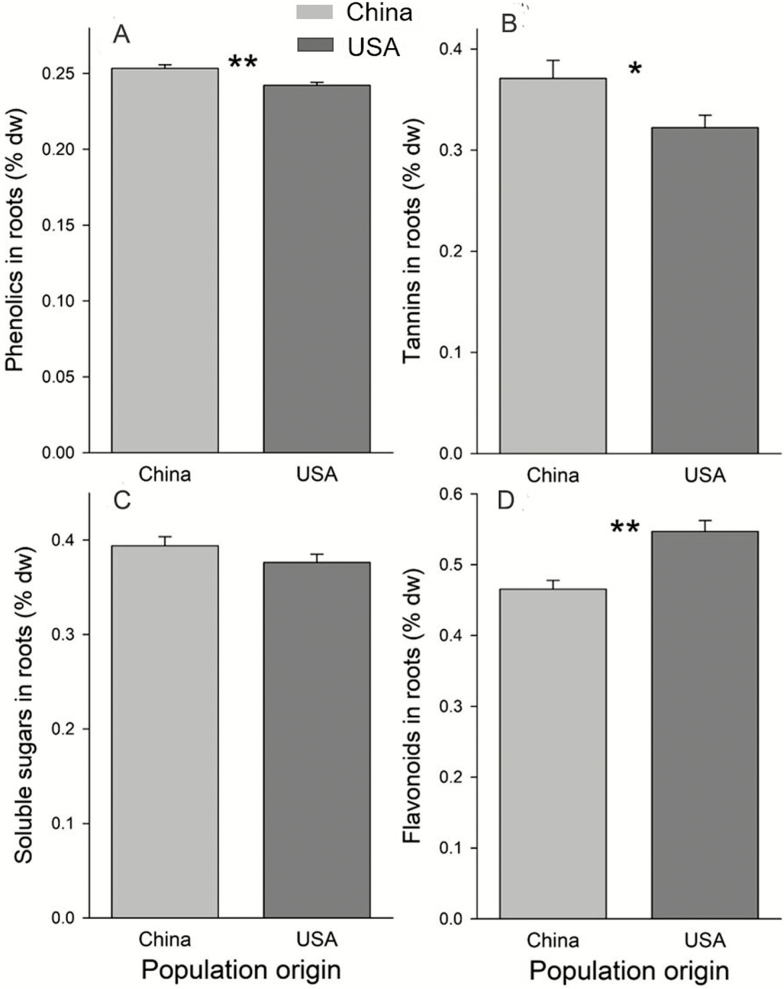
Concentrations of (A) phenolics, (B) tannins, (C) soluble sugars and (D) flavonoids in roots of *T. sebifera* plants from native (China) or introduced (USA) populations. Difference of naive (China) and introduced (USA): *P < 0.05; **P < 0.01.

Compared to plants from native populations, those from introduced populations had lower root concentrations of phenolics (Fig. 2A; *F*_1, 15_ = 11.08, *P* = 0.0046) and tannins (Fig. 2B; *F*_1, 15_ = 5.03, *P* = 0.0405), comparable concentrations of soluble sugars (Fig. 2C; *F*_1, 15_ = 1.87, *P* = 0.1919) and higher concentrations of flavonoids (Fig. 2D; *F*_1, 15_ = 10.81, *P* = 0.0050). None of these chemical concentrations varied with population (all *P* > 0.10).

### The relationship between flavonoids and AMF

**Figure 3.**
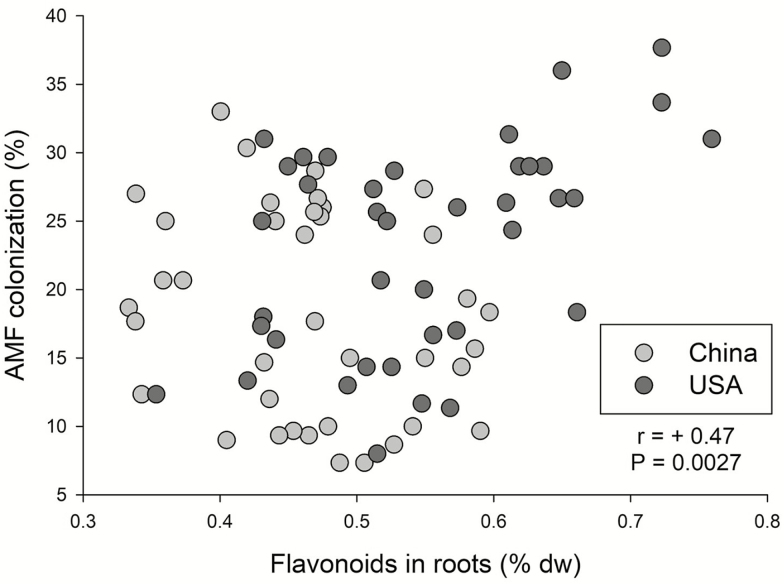
The relationship between root flavonoids and AMF colonization. The *r* and *P*-values are from a Pearson correlation with all plants.

**Figure 4.**
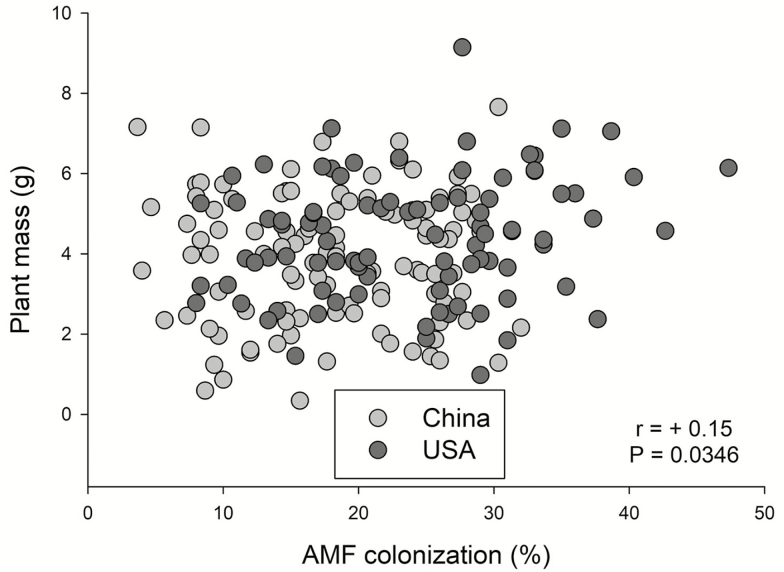
The relationship between AMF colonization and plant mass. The *r* and *P*-values are from a Pearson correlation with all plants.

AMF colonization was significantly positively correlated with the concentration of root flavonoids (Fig. 3; *r* = +0.47, *P* = 0.0027) but was not related to concentrations of phenolics (*P* = 0.5017), tannins (*P* = 0.7394) or soluble sugars (*P* = 0.7252). When introduced and native populations were examined separately, the correlation between root flavonoids and AMF colonization was significant for introduced populations (*r* = +0.48, *P* = 0.0027) but not native populations (*P* = 0.17). Plant mass and AMF colonization rate were positively correlated (Fig. 4; *r* = +0.15, *P* = 0.0346).

## Discussion

An increasing number of studies support an important role for symbiotic micro-organisms in plant invasions (Dawson and Schrama 2016; van Kleunen *et al.* 2018), however, we know little about how invasive plants enhance their interaction with AMF. Yet, knowing the mechanisms that regulate these symbiotic relationships would help to understand, predict and manage plant invasions. In this study, we found that introduced populations of *T. sebifera* enhanced their symbiotic relationship with AMF apparently by having higher levels of root flavonoids. That in turn may increase their growth ability and competitiveness in the introduced range. To the best of our knowledge, this study is the first to report such a linkage between root secondary chemicals, AMF and invasive plant growth by comparing the differences between plants from the introduced and native populations.

### Variation of AMF relationships between population origins

Previous studies have reported that *T. sebifera* plants from the introduced range have higher AMF colonization rates than those from the native range, which is consistent with our results (Yang *et al.* 2013, 2015c). As was the case in these other studies, we also found that AMF colonization and plant growth were positively related. Although we did not examine other benefits of AMF colonization, they have been associated with higher levels of tolerance to soil salinity and water stress (Yang *et al.* 2015a, b), which may help to explain the high tolerance of *T. sebifera* to these stressful conditions in the introduced range (Howard 2012; Paudel and Battaglia 2013, 2015).

Our results indicated that *T. sebifera* plants are able to increase AMF associations and benefit their success as has been shown for other invasive plants. For example, the invasive plants, *Agropyron cristatum*, *C. stoebe* and *E. esula* may increase the abundance and diversity of AMF communities and accelerate their invasions (Reinhart *et al.* 2017). In addition, activated carbon applications to invasive populations of *S. canadensis* limit their selective enhancement of beneficial AMF (Yuan *et al.* 2014) and support a role for root secondary chemicals in this enhancement. This is in agreement with our finding that flavonoids enhance AMF colonization. Although soil hyphae likely include many of fungal types other than AMF, our finding that hyphae were abundant in soil associated with plants from introduced populations supports a role for root exudates in shaping the soil microbial community (Siegrid *et al.* 2007).

### Flavonoids were positively correlated with AMF colonization

Previous studies have shown that secondary metabolites in plants play a role in regulating AMF symbiosis (Kohki *et al.* 2005; Kikuchi *et al.* 2007; Barto *et al.* 2010), such as flavones, phenolics and saponins which are three general types of secondary metabolites in many plants (Scervino *et al.* 2007), that can accumulate in soil (Zhang *et al.* 2011). Phenolics may reduce AMF colonization (Piotrowski *et al.* 2008), but flavones (e.g. quercetin and luteolin) may enhance AMF symbiosis (Siegrid *et al.* 2007). In this study, we found that introduced populations of *T. sebifera* had lower levels of root phenolics and tannins but higher flavonoids indicating that introduced populations may have genetic traits that enhance AMF colonization, which is consistent with the finding that flavonoids increasing the rhizobium nodules in legumes (Liu *et al.* 2017). Additionally, AMF could regulate the nodulation of rhizobium (Jin *et al.* 2018; Girardin *et al.* 2019). The positive correlation we found between flavonoids and AMF supports a higher overall level of root flavonoids as driving higher AMF colonization. But the distinct relationships of flavonoids and AMF colonization for introduced vs. native populations suggest that the flavonoid chemical composition may also vary between population origins and contribute to variation in AMF colonization. However, it is unclear which specific flavonoid chemicals have the main regulatory effect on AMF colonization for *T. sebifera* or other invasive plants.

## Conclusions

Many studies have examined the secondary compounds in introduced vs. native populations of invasive plants but these have largely focused on anti-herbivore defences. Here we found evidence that *T. sebifera* plants may produce more root flavonoids or different types of root flavonoids which increases their AMF associations and in turn their rapid growth. Because there are many types of flavonoids, future studies should examine the variation in individual flavonoids and test their effects on spore germination and hyphal growth of AMF in the introduced range. Because our study did not experimentally manipulate root chemicals this would strengthen the inferences regarding their effects on AMF colonization. In addition, it is critical to understand how differences in secondary chemicals in *T. sebifera* and native plants in the introduced range may influence the plant invasive success, especially with mycorrhizal associations being more beneficial to *T. sebifera* than native tree species with which it co-occurs (Nijjer *et al.* 2004). Indeed, understanding the traits of introduced plants that make them likely to have enhanced benefits from mycorrhizae in the introduced range compared to native plants with which they compete is critical for predicting and managing plant invasions.

## Supporting Information

The following additional information is available in the online version of this article—

**Supplement.** Supporting information for DATA.

**Supplement S2.** Supporting information for data analysis CODE.

plaa002_suppl_Supplementary-Material-1

plaa002_suppl_Supplementary-Material-2

## Data

The complete data for the analyses are also available as **Supporting Information**.

## Sources of Funding

This work was supported by the National Natural Science Foundation of China (31600300, 31971558, and 31770414) and the National Key Research and Development Program (YFC20171200100).

## Contributions by the Authors

J.D. designed this experiment and revised the manuscript; Y.P. and B.T. carried out the experiment; E.S. did the data analysis; B.T. drafted the manuscript; J.D. and E.S. edited the manuscript. All authors read and approved the final manuscript.

## Conflict of Interest

There is no any conflict of interest in this study.
